# Preparation of Comb-Shaped Polyether with PDMS and PEG Side Chains and Its Application in Polymer Electrolytes

**DOI:** 10.3390/molecules30153201

**Published:** 2025-07-30

**Authors:** Tomoya Enoki, Ryuta Kosono, Nurul Amira Shazwani Zainuddin, Takahiro Uno, Masataka Kubo

**Affiliations:** Division of Applied Chemistry, Graduate School of Engineering, Mie University, 1577 Kurimamachiya-cho, Tsu 514-8507, Japan; 424d001@m.mie-u.ac.jp (T.E.); 423m322@m.mie-u.ac.jp (R.K.); 422de02@m.mie-u.ac.jp (N.A.S.Z.); uno@chem.mie-u.ac.jp (T.U.)

**Keywords:** solid polymer electrolyte, comb-shaped polyether, grafting, hydrosilylation, click chemistry, ionic conductivity

## Abstract

Polyethylene oxide (PEO) is the most well-studied polymer used in solid polymer electrolytes (SPEs) for lithium ion batteries (Li-ion batteries). However, ionic conductivity is greatly reduced in the low temperature range due to the crystallization of PEO. Therefore, methods to suppress the crystallization of PEO at room temperature by cross-linking or introducing a branched structure are currently being investigated. In this study, we synthesized new comb-type ion-conducting polyethers with two different side chains such as polydimethylsiloxane (PDMS) and polyethylene glycol monomethyl ether (mPEG) segments as flexible and ion-conducting segments, respectively. The introduction of the PDMS segment was found to prevent a decrease in ionic conductivity in the low-temperature region, but led to an ionic conductivity decrease in the high temperature region. On the other hand, the introduction of mPEG segments improved ionic conductivity in the high-temperature region. The introduction of mPEG segments with longer chains resulted in a significant decrease in ionic conductivity in the low-temperature region.

## 1. Introduction

A rechargeable battery is a type of electrical battery which can be charged, discharged into a load, and recharged many times. The first rechargeable battery was a lead–acid battery, which was commercialized around 1900. Then, a nickel–cadmium battery was put into practical use around 1950, and this nickel–cadmium battery attracted attention around 1980 for use in portable equipment, and the sole power source until around 1995. Nickel–metal hydride batteries became available in 1989. The battery has a hydrogen-absorbing alloy for the negative electrode instead of cadmium. The nickel–cadmium battery was replaced by the nickel–metal hydride battery, which has a higher energy density than the nickel–cadmium battery and a voltage of 1.2 V, the same as that of the nickel–cadmium battery. Since cadmium is a toxic element, nickel–cadmium batteries were banned for most uses by the European Union in 2004.

Rechargeable lithium-ion batteries were commercialized in 1991. Lithium-ion batteries are high-performance rechargeable batteries with high energy density and high output, and have applications not only in small-sized portable electronic devices such as laptop computers and cellular phones, but also in large-sized applications such as electric vehicles. The active material for the positive electrode is a transition metal oxide lithium compound such as LiCoO_2_, and the active material for the negative electrode is a carbonaceous material such as graphite. The electrolyte is usually a lithium salt dissolved in a mixture of organic carbonates. Although lithium-ion batteries are in commercial production, much research is still being carried out today to achieve higher performance [1,2]. A problem for conventional lithium-ion batteries is the usage of flammable organic solvents in the electrolyte, which poses the risk of leakage, ignition, and decomposition of the solvent during the charging and discharging process.

All-solid-state electrolytes are attracting much attention for fabricating safe lithium-ion batteries that do not ignite. Candidates for solid electrolytes can be classified into inorganic electrolytes such as oxides and sulfides and polymer electrolytes. Polymer electrolytes are characterized by their light weight and flexibility, and are expected to have applications in wearable devices. Since Wright [3] and Armand [4] found that alkali metal ions can migrate in polyethylene oxide (PEO), PEO has been the most well-studied polymer for solid polymer electrolytes (SPEs) [5,6,7,8,9,10]. The mechanism of ionic conduction in PEO is thought to be the coordination of metal ions to ether oxygen followed by movement along the segmental motion of the polymer chain [11]. Since PEO is prone to crystallization due to its highly symmetric structure, ionic conductivity decreases greatly below its melting point. Various attempts have been made to suppress the crystallization of PEO to prevent ionic conductivity decrease in low-temperature regions, such as by the addition of a low molecular weight of liquid organic compounds as a plasticizer [12], the addition of an inorganic filler such as silica, alumina and titanium dioxide [13,14], polymer blending [15] and the modification of chain architecture [16]. We prepared polypseudorotaxane composed of linear and cyclic PEOs to find that the formation of polypseudorotaxane played an important role in crystallization suppression [17]. Wu and Shen reported a polymer electrolyte based on ethylene oxide (EO) and propylene oxide (PO) copolymer (P(EO/PO)) [18]. They found that the incorporation of an appropriate amount of PO unit into the polymer chains increased the ionic conductivity of the P(EO/PO) complex by interrupting long EO segments. The combination of PEO with a flexible polymer such as polydimethylsiloxane (PDMS) is also an attractive strategy for the suppression of PEO crystallization. The main chain of PDMS consists of repeating Si-O bonds. The Si-O bonds have longer bond distances than C-C bonds, and the rotational barrier energy around the bond axis is almost zero. Therefore, PDMS is a polymer with extremely flexible properties. Trapa et al. reported a microphase-separating single-ion conductor, poly[methacrylate-ran-lithium methacrylate]-graft-PDMS, which was prepared by lithiating a precursor polymer synthesized by free radical methods using commercially available macromonomers [19]. Lee et al. synthesized polymer electrolytes based on polyethylene glycol methacrylate-graft-PDMS to improve ionic conductivity in the low-temperature region [20]. A blend-type polymer electrolyte composed of PEO, PDMS, and lithium hexafluorophosphate (LiPF_6_) was reported by Das et al., who found that ionic conductivity in the low-temperature region was improved [21]. Wakler reported on tunable networks from thiolene chemistry for lithium ion conduction prepared by the cross-linking of end-functionalized poly (ethylene glycol) (PEG) and PDMS [22].

**Figure 1:**
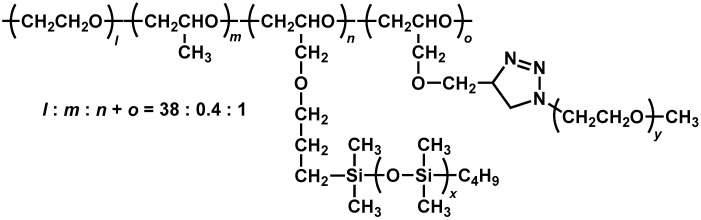
Chemical structure of PDMS & mPEG-g-P(EO/PO/AGE) studied in this work.

We are interested in a terpolymer composed of EO, PO, and allyl glycidyl ether (AGE) (P(EO/PO/AGE)) because it is possible to modify P(EO/PO/AGE) to a comb-shaped polymer by using the reaction of the allyl group. Most comb polymers so far utilize polyethylene glycol acrylate or methacrylate. To the best of our knowledge, there are few reports regarding comb-shaped polymer solid electrolytes which carry two different branch polymers. We introduced both PDMS chains and polyethylene glycol monomethyl ether (mPEG) chains onto P(EO/PO/AGE) to obtain PDMS & mPEG grafted P(EO/PO/AGE) (PDMS & mPEG-g-P(EO/PO/AGE)) (Figure 1) and examined their ionic conductivity after complexation with lithium bis (trifluoromethanesulfonyl) imide (LiTFSI) as a lithium salt.

## 2. Results and Discussion

### 2.1. Preparation and Properties of PDMS-g-P(EO/PO/AGE)

In this study, two kinds of branch polymers, such as PDMS and mPEG, were introduced onto P(EO/PO/AGE). Our choice was to introduce PDMS chains first and then mPEG chains as the order of introducing the branch polymers. The reason for this sequence was to consider molecular weights of PDMS (MW = 850) and mPEGs (MW = 1000, 2000, and 4000), because introducing mPEG chains with high molecular weight first makes it difficult for PDMS chains to react with allyl groups remaining on the main chain due to steric hindrance of the mPEG segment.

**Figure 2:**
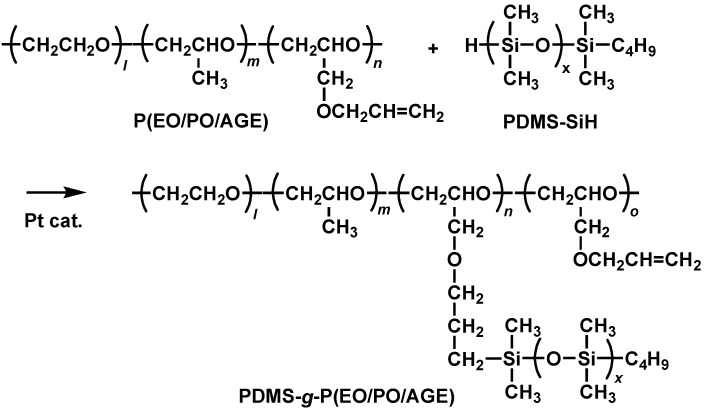
Preparation of PDMS-*g*-P(EO/PO/AGE) by hydrosilylation reaction.

**Figure 3:**
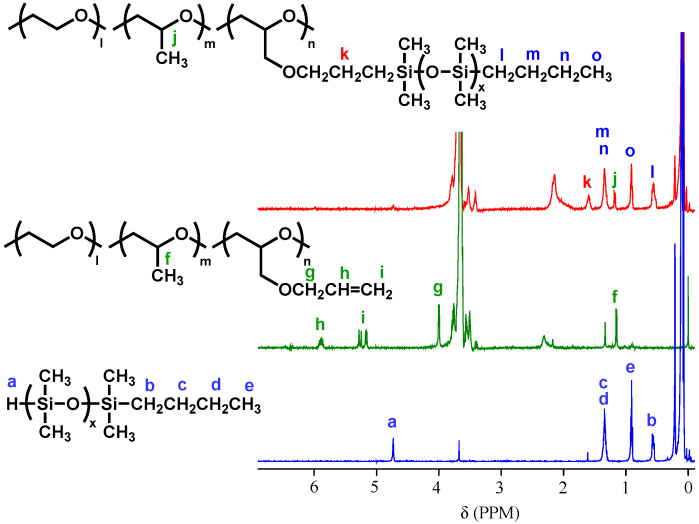
^1^H NMR spectra of (blue) PDMS-SiH, (green) P(EO/PO/AGE) and (red) PDMS-*g*-P(EO/PO/AGE).

The introduction of PDMS chains onto P(EO/PO/AGE) was carried out through a hydrosilylation reaction between mono hydride-terminated PDMS (PDMS-SiH) and P(EO/PO/AGE) in the presence of a platinum catalyst (Figure 2). The introduction percentage of PDMS chains was adjusted by changing the amount of PDMS-SiH relative to the moles of allyl groups. Figure 3 shows the ^1^H NMR of PDMS-*g*-P(EO/PO/AGE) with a 100% introduction of PDMS together with those of P(EO/PO/AGE) and PDMS-SiH. After reaction, the signal due to Si-H groups at 4.7 ppm and the allyl group signals at 5.2 and 5.8 ppm disappeared completely. In addition, the absorption signals due to the terminal butyl group of PDMS were clearly observed (peaks l, m, n, and o). This indicates that the grafting reaction proceeded successfully, resulting in a comb-shaped polymer with PDMS chains bonded to all allyl groups.

**Figure 4:**
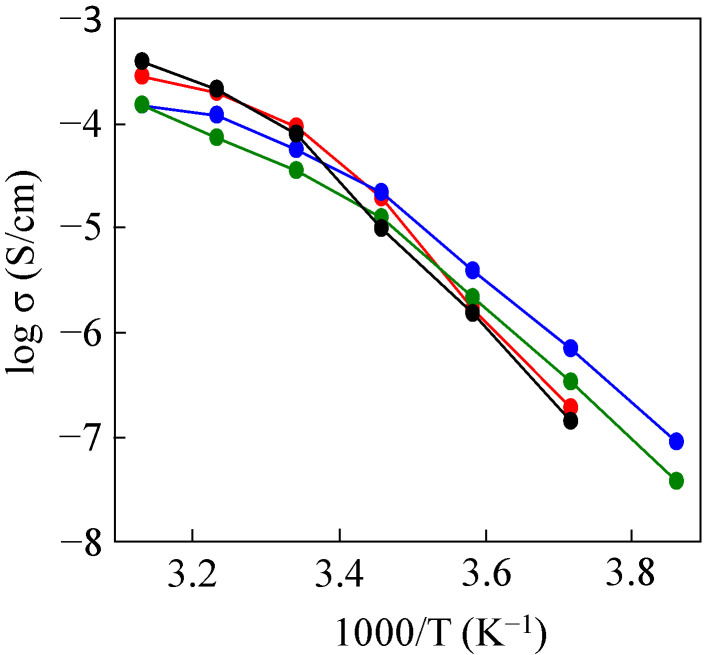
Temperature dependences of ionic conductivities for SPEs based on P(EO/PO/AGE) (black), 25% PDMS-*g*-P(EO/PO/AGE) (red), 50%PDMS-*g*-P(EO/PO/AGE) (green), and 75%PDMS-g-P(EO/PO/AGE) (blue).

Figure 4 shows the ionic conductivity for solid polymer electrolytes (SPEs) based on P(EO/PO/AGE) and PDMS-*g*-P(EO/PO/AGE) with 25%, 50%, and 75% introductions of PDMS using lithium bis (trifluoromethanesulfonyl) imide (LiTFSI) as a lithium salt ([LiTFSI]/[EO] = 1/15). In each case, ionic conductivity increased with increasing temperature, as is typical for PEO-based amorphous polymer electrolytes, and showed convex curves. The ionic conductivity of P(EO/PO/AGE) decreased significantly below 30 °C (1000/T~3.3) due to the crystallization of the polyether main chain (black). The effect of the introduced PDMS chains was different in high- and low-temperature regions (red). PDMS chains prevented large conductivity decreases, probably due to the suppression of the crystallization of the main chain. On the other hand, the ionic conductivity of the electrolyte based on PDMS-g-P(EO/PO/AGE) was about an order of magnitude lower than that based on P(EO/PO/AGE) in the high-temperature region. In the high-temperature region, the polyether main chain can move without problems, so PDMS chains that are not ion-conducting are not good for ionic conduction. Therefore, we introduced additional mPEG chains onto the main chain polyether to improve ionic conductivity.

**Figure 5:**
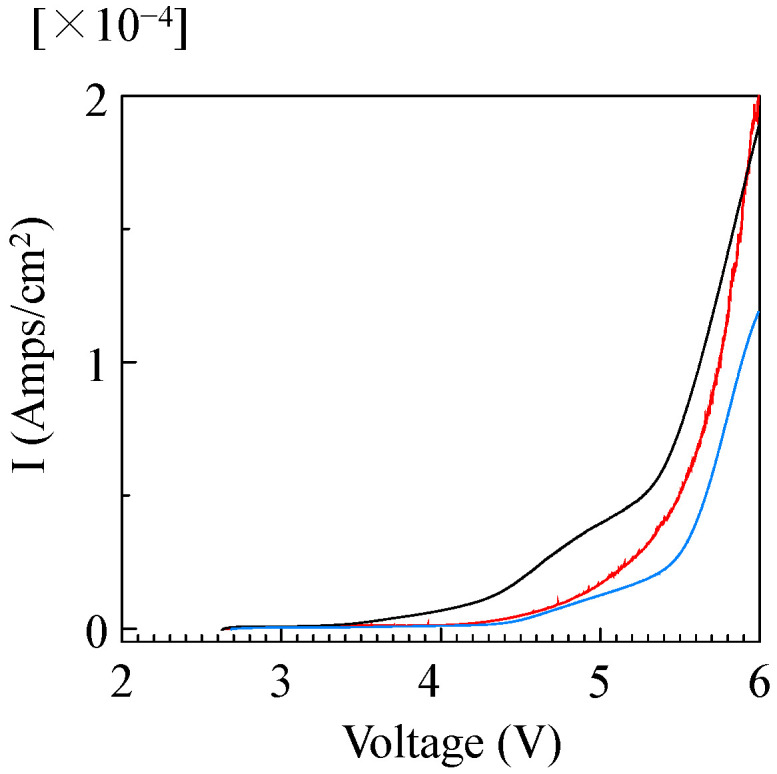
Linear sweep voltammetry curves for SPEs based on (black) P(EO/PO/AGE), (blue) 50%PDMS-*g*-P(EO/PO/AGE), and (red) 75%PDMS-*g*-P(EO/PO/AGE) containing LiTFSI ([LiTFSI]/[EO] = 1/15); scan rate, 10 mVs^−1^; temperature, 20 °C.

Electrochemical stability (oxidation stability) for SPEs based on PDMS-*g*-P(EO/PO/AGE)s was evaluated by measuring linear sweep voltammetry, as shown in Figure 5. The breakdown voltages (vs Li/Li^+^) for SPEs based on P(EO/PO/AGE), 50%PDMS-*g*-P(EO/PO/AGE), and 75%PDMS-*g*-P(EO/PO/AGE) were 3.9, 4.3, and 4.3 V, respectively. It was found that the introduction of the PDMS segment onto the main chain improved the oxidation stability.

### 2.2. Preparation of PDMS & mPEG-g-P(EO/PO/AGE)

**Figure 6:**
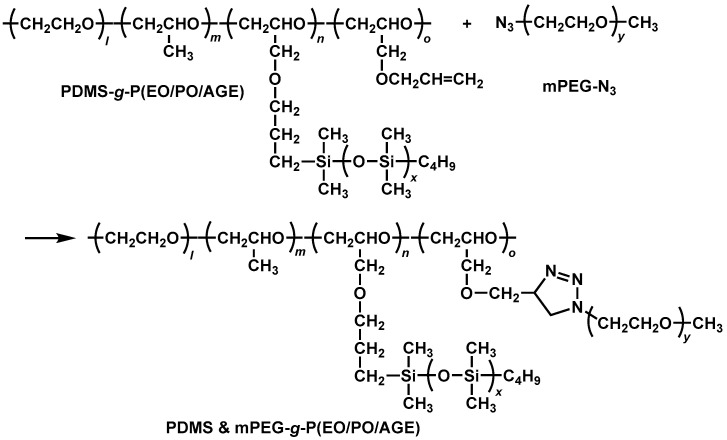
Preparation of PDMS & mPEG-g-P(EO/PO/AGE).

We synthesized three PDMS-*g*-P(EO/PO/AGE) with 25, 50, and 75% of introduction of PDMS chains to the allyl group. Then, we carried out a thermal allyl-azide click reaction [23] of PDMS-*g*-P(EO/PO/AGE)s with azide-terminated mPEG (mPEG-N_3_) with a molecular weight of 1000 (mPEG_1K_-N_3_), 2000 (mPEG_2K_-N_3_), and 4000 (mPEG_4K_-N_3_) to obtain nine PDMS & mPEG-g-P(EO/PO/AGE)s in total (Figure 6). For example, a polymer with 50% PDMS introduced to the allyl groups and mPEG with molecular weight of 1000 introduced to the remaining 50% of the allyl groups is denoted as 50%PDMS & 50%mPEG_1K_-*g*-P(EO/PO/AGE).

**Figure 7:**
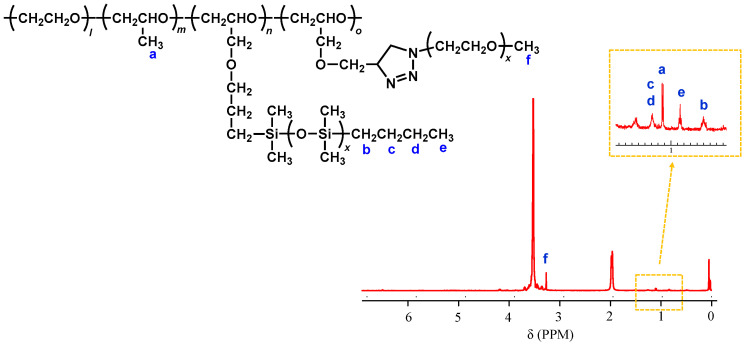
^1^H NMR spectrum of 50%PDMS & 50%mPEG_2K_-*g*-P(EO/PO/AGE).

Figure 7 shows the ^1^H NMR of the resulting comb-shaped polymer, 50%PDMS & 50%mPEG_2K_-*g*-P(EO/PO/AGE), as an example. The absorption peaks originating from the allyl groups have completely disappeared, indicating that mPEG segments have been introduced to all of the remaining allyl groups. In addition, the absorptions (from a to f) shown in the figure are characteristic of the two branch polymers, and the integral ratios confirm that the two polymer chains were introduced in the same proportion as the feed composition.

**Figure 8:**
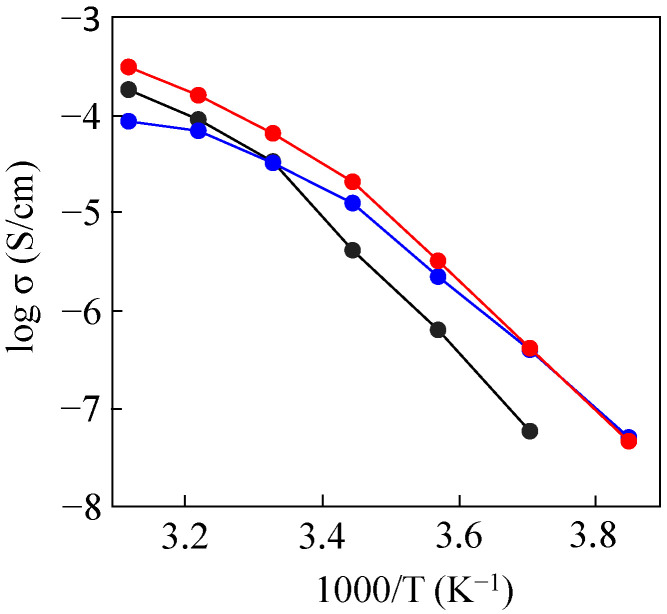
Temperature dependences of ionic conductivities for the SPEs based on P(EO/PO/AGE) (black), 50%PDMS-*g*-P(EO/PO/AGE) (blue), and 50%PDMS & 50%mPEG_2K_-g-P(EO/PO/AGE) (red).

Figure 8 shows ionic conductivities of SPEs based on P(EO/PO/AGE), 50%PDMS-*g*-P(EO/PO/AGE), and 50%PDMS & 50%mPEG_2K_-*g*-P(EO/PO/AGE). As already mentioned, the introduction of PDMS segments improved ionic conductivity in the low-temperature region, but decreased ionic conductivity in the high-temperature region. The additional introduction of ion-conducting mPEG segments further improved ionic conductivity in the high-temperature region without decreasing ionic conductivity in the low-temperature region. For example, at 50 °C, the ionic conductivity of SPE based on 50%PDMS & 50%mPEG_2K_-*g*-P(EO/PO/AGE) (3.2 × 10^−4^ S/cm) was about three times higher than that based on 50%PDMS-*g*-P(EO/PO/AGE) (7.9 × 10^−5^ S/cm).

**Figure 9:**
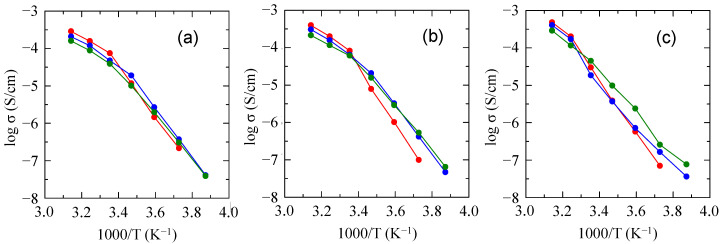
Ionic conductivity of SPEs based on (**a**) PDMS & mPEG_1K_-*g*-P(EO/PO/AGE), (**b**) PDMS & mPEG_2K_-*g*-P(EO/PO/AGE), and (**c**) PDMS & mPEG_4K_-*g*-P(EO/PO/AGE) with various PDMS/mPEG ratios (red: 25%PDMS & 75%mPEG; blue: 50%PDMS & 50%mPEG; green: 75%PDMS & 25%mPEG).

Figure 9 shows the effects of the PDMS/mPEG ratio and molecular weight of mPEG on the ionic conductivity of SPEs based on nine PDMS-*g*-P(EO/PO/AGE)s. Regardless of the molecular weight of mPEG, polymers with lower PDMS content exhibited higher ionic conductivity in the high-temperature region. On the other hand, in the low-temperature region, the ionic conductivity was higher for the polymer with higher PDMS content. This is probably due to the fact that the PDMS segments, which do not contribute to ion conduction in the high-temperature region, suppressed crystallization of the polyether in the low-temperature region, as described before.

**Figure 10:**
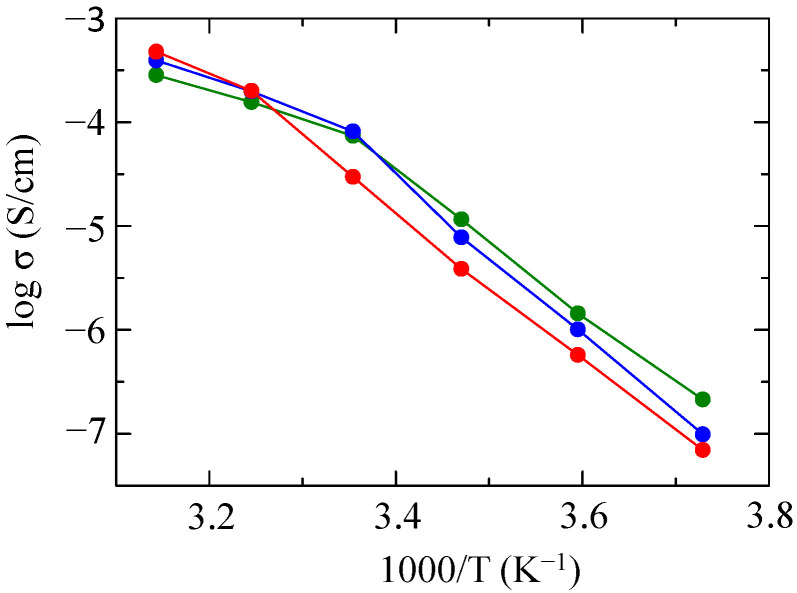
Temperature dependences of ionic conductivities for the SPEs based on 25%PDMS & 75%mPEG_1K_-*g*-P(EO/PO/AGE) (green), 25%PDMS & 75%mPEG_2K_-*g*-P(EO/PO/AGE) (blue), and 25%PDMS & 75%mPEG_4K_-*g*-P(EO/PO/AGE) (red).

We examined the effect of the molecular weight of mPEG on ionic conductivity. As a representative example, Figure 10 shows the ionic conductivities of three SPEs (25%PDMS & 75%mPEG) with different molecular weights of mPEG. As the molecular weight of mPEG increased, the ionic conductivity increased in the high-temperature region, but conversely, in the low-temperature region, the ionic conductivity decreased with an increase in the molecular weight of mPEG.

**Figure 11:**
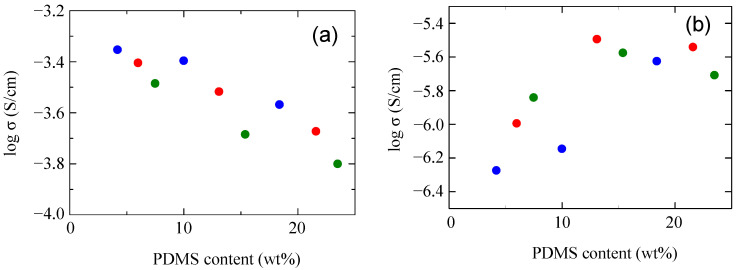
Relationship between ionic conductivity and PDMS content in the comb-shaped polymers with various molecular weights of mPEG (green: 1000; red: 2000; blue: 4000) at (**a**) 45 °C and (**b**) 5 °C.

To clarify the effect of the mPEG chain length, ionic conductivity in the high-temperature region at 45 °C and in the low-temperature region at 5 °C were examined. Figure 11 shows the relationships between ionic conductivity and PDMS content in the comb-shaped polymers with three different molecular weights of mPEG. The effect of the molecular weight of mPEG was completely different at 45 °C and 5 °C. At 45 °C, the ionic conductivity increased with the molecular weight of mPEG. On the other hand, at 5 °C, the ionic conductivity decreased with the molecular weight of mPEG, and when the molecular weight reached 4000, the ionic conductivity decreased significantly.

Table 1 summarizes PDMS content, crystallization temperature (*T*_c_), and melting temperature (*T*_m_) for nine PDMS & mPEG-g-P(EO/PO/AGE)s. It was found that if the molecular weight of mPEG is the same, PDMS content did not have significant effect on the crystallization temperature or melting point. For example, when the molecular weight of mPEG is 1000, the *T*_c_ of the comb-shaped polymer was 33, 32 and 31 °C for 25%PDMS & 75%mPEG-g-P(EO/PO/AGE), 50% PDMS & 50%mPEG-g-P(EO/PO/AGE) and 75%PDMS & 25%mPEG-g-P(EO/PO/AGE), respectively. The *T*_m_ was 50, 51, and 49 °C for 25%PDMS & 75%mPEG-g-P(EO/PO/AGE), 50% PDMS & 50%mPEG-g-P(EO/PO/AGE), and 75%PDMS & 25%mPEG-g-P(EO/PO/AGE), respectively. Similar results were obtained when the molecular weight of mPEG was 2000 and 4000. On the other hand, the molecular weight of mPEG introduced as the second branch moiety had a significant effect on the crystallization temperature and melting point. As the molecular weight increased from 1000 to 2000 to 4000, the crystallization temperature and melting point increased, resulting in easy crystallization. In other words, the decrease in ionic conductivity in the low-temperature region was attributed to crystallization of the mPEG at the branch sites.

## 3. Materials and Methods

### 3.1. Reagents

P(EO/PO/AGE) (EO:PO:AGE = 38:0.4:1, MW = 61,000) was a gift from Meisei Chemical Works, Ltd. (Kyoto, Japan). α-Azide, ω-methoxy heterodifunctional polyethylene glycols (mPEG-N_3_) were prepared according to the reported procedure [24,25]. Mono-hydride polydimethylsiloxane (MW = 850) (PDMS-H, MCR-H07) and platinum-divinyltetramethyldisiloxane complex (3.0% Pt in vinyl-terminated PDMS) (SIP 6830) were purchased from Gelest, Inc. (Morrisville, PA, USA). All other reagents were obtained from commercial sources and used as received.

### 3.2. Preparation of α-p-Toluenesulfonyloxy, ω-Methoxy Heterodifunctional PEG

In a typical example, into a solution of polyethylene glycol monomethyl ether (mPEG_2K_) (MW = 2000) (10 g, 5 mmol), triethylamine (0.76 g, 7.5 mmol), and 4-dimethylaminopyridine (DMAP) (92 mg, 0.75 mmol) in dichloromethane (40 mL) was added dropwise 1.43 g (7.5 mmol) of *p*-toluenesulfonyl chloride dissolved in 15 mL of dichloromethane and the reaction mixture was stirred at room temperature for 48 h. The reaction mixture was diluted with dichloromethane and washed successively with 1N HCl, 5% NaOH, and distilled water. The organic layer was dried over anhydrous magnesium sulfate, concentrated under reduced pressure, and poured into large amount of ether to precipitate 9.7 g (90%) of α-*p*-toluenesulfonyloxy, ω-methoxy heterodifunctional PEG_2K_ as a white powder; ^1^H NMR (400 MHz, CDCl_3_, δ): 7.77 (d, *J* = 8.2 Hz), 7.32 (d, *J* = 8.2 Hz), 4.13 (t, *J* = 5.0 Hz), 3.7–3.5 (m), 3.36 (s), 2.43 (s); ^13^C NMR (100 MHz, CDCl_3_, δ): 144.7, 132.9, 129.7, 127.9, 71.9, 70.7, 69.2, 68.6, 59.0); IR (NaCl, cm^−^^1^): 2870, 1108.

### 3.3. Preparation of α-Azide, ω-Methoxy Heterodifunctional PEG (mPEG-N_3_)

In a typical example, a mixture of α-*p*-toluenesulfonyloxy, ω-methoxy heterodifunctional PEG_2K_ (MW = 2150) (9.6 g, 4.5 mmol) and sodium azide (0.88 g, 13.5 mmol) in 30 mL of acetonitrile was heated under reflux for 12 h. The reaction mixture was poured into distilled water and extracted with dichloromethane three times. The combined organic layer was dried over anhydrous magnesium sulfate, concentrated under reduced pressure, and poured into a large amount of ether to precipitate 8.6 g (96%) of mPEG_2K_-N_3_ as a white powder; ^1^H NMR (400 MHz, CDCl_3_, δ):3.6 (m), 3.38 (s); ^13^C NMR (100 MHz, CDCl_3_, δ):70.6, 58.2, 50.7; IR (NaCl, cm^−1^): 2870, 2110, 1108.

### 3.4. Preparation of PDMS-g-P(EO/PO/AGE)

In a typical example, into a solution of P(EO/PO/AGE) (EO:PO:AGE = 38:0.4:1, MW = 61,000) (6.0 g, CH_2_=CHCH_2_ = 3.3 mmol) and PDMS-SiH (MW = 850) (1.4 g, 1.7 mmol) in 50 mL of benzene was added 20 mg of SIP 6830 catalyst, and the mixture was heated at 90 °C for 48 h under nitrogen. The reaction mixture was poured into hexane to precipitate the polymer to obtain 7.0 g (95%) of 50%PDMS-*g*-P(EO/PO/AGE) as a light gray elastic solid. Table 2 summarizes reaction conditions for PDMS-*g*-P(EO/PO/AGE)s.

### 3.5. Preparation of PDMS & PEG-g-(EO/PO/AGE)

In a typical example, a mixture of 50%PDMS-*g*-P(EO/PO/AGE) (1.0 g, CH_2_=CHCH_2_ = 0.22 mmol) and mPEG_2K_-N_3_ (0.48 g, 0.24 mmol) in 20 mL of toluene was heated under reflux for 48 h. The reaction mixture was poured into ether to precipitate the polymer to obtain 1.2 g (83%) of 50%PDMS & 50%mPEG_2K_-*g*-P(EO/PO/AGE) as a white solid. Table 3 summarizes the reaction conditions for the preparation of PDMS & mPEG-g-P(EO/PO/AGE)s.

### 3.6. Preparation of Solid Polymer Electrolyte

**Figure 12:**
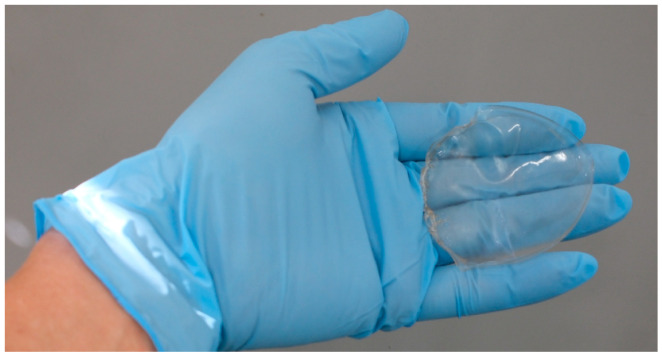
Photograph of SPE based on 50%PDMS & 50%mPEG_2K_-*g*-P(EO/PO/AGE).

A given amount of polymer and LiTFSI were dissolved in a small amount of acetonitrile ([LiTFSI]:[CH_2_CH_2_O] = 1:15). The resulting solution was put into a Teflon Petri dish and the solvent was progressively evaporated at 50 °C. Finally, the obtained film was allowed to dry under reduced pressure at 80 °C for 24 h. All preparation procedures were carried out in an argon-filled glovebox. Figure 12 shows a photograph of SPE based on 50%PDMS & 50%mPEG_2K_-*g*-P(EO/PO/AGE) ([LiTFSI]:[CH_2_CH_2_O] = 1:15). It was a transparent and flexible solid.

### 3.7. Ionic Conductivity Measurement

Ionic conductivities of the SPEs were measured by the two-probe method after the samples had been fixed inside a Teflon O-ring spacer with known thickness and sandwiched between two stainless steel (SS) electrode disks acting as ion-blocking electrodes and set in a thermostat oven chamber. The measurements were carried out using a Solartron 1260 impedance/gain-phase analyzer over a frequency range of 10^6^ to 1 Hz and a temperature range of 80 to 10 °C with an amplitude of 10 mV. All samples were first kept at 80 °C for at least 12 h and then measured during the cooling cycle. The measurements were carried out after keeping the samples at each temperature for 1 h to attain thermal equilibration. The data were processed by using an appropriate fitting program.

### 3.8. Instrumentation

^1^H NMR spectra were recorded at room temperature on a JEOL JNM-ECZ 400 nuclear magnetic resonance spectrometer. Samples were dissolved in deuterated chloroform (CDCl_3_) and tetramethylsilane (TMS) was used as the internal standard. Gel permeation chromatography (GPC) was carried out on a Tosoh HLC-8020 chromatograph equipped with polystyrene gel columns (Tosoh TSK gel Multipore HXL-M; exclusion limit 2 × 10^6^; 300 × 7.5 mm) and refractive/ultraviolet dual mode detectors. Tetrahydrofuran (THF) was used as the eluent at a flow rate of 1.0 mL/min. The calibration curves for GPC analysis were obtained using polystyrene standards. Differential scanning calorimetry (DSC) measurement was carried out on an SII EXSTER 6000 thermal analysis instrument DSC 6220 under nitrogen gas flow at a heating rate of 10 K/min. About 10 mg of the sample was weighted, loaded in an aluminum pan, and then sealed. The samples were firstly heated at 100 °C and secondly cooled to −100 °C to observe crystallization temperature (*T*_c_). Lastly, the samples were heated again to observe melting temperature (*T*_m_).

## 4. Conclusions

We prepared novel comb-shaped polyethers with two different kinds of branch polymer chains such as PDMS and mPEG. PDMS and mPEG chains are flexible and ion conducting segments, respectively. First, we carried out hydrosilylation reaction of terpolymer composed of EO, PO, and AGE with mono hydride-terminated PDMS to obtain PDMS-*g*-P(EO/PO/AGE). Second, mPEG chains were introduced as the second branch component by the thermal allyl-azide click reaction between PDMS-g-P(EO/PO/AGE) and azide-terminated mPEG to obtain nine PDMS & mPEG-g-P(EO/PO/AGE)s with different PDMS/mPEG ratios and molecular weights of mPEG. Finally, we prepared SPEs by complexation of these comb-shaped polyethers with LiTFSI. When the ratio of PDMS chains to mPEG chains was changed to 75:25, 50:50, and 25:75, the ionic conductivity in the high-temperature region decreased as the ratio of PDMS chains increased, but the ionic conductivity in the low-temperature region increased. On the other hand, when the molecular weight of the PEG chains was changed to 1000, 2000, and 4000, the ionic conductivity in the high-temperature region improved with increasing mPEG chain length. Meanwhile, in the low-temperature region, the ionic conductivity increased as the molecular weight of the mPEG chain increased from 1000 to 2000, but decreased as the molecular weight of the mPEG chain increased to 4000. These experimental results indicate that crystallization of mPEG chains has a significant effect on ionic conductivity. Our present research is based on a terpolymer, P(EO/PO/AGE) (EO:PO:AGE = 38:0.4:1), which contains relatively small amounts of allyl groups. In the future, we plan to investigate comb-shaped polymers with more densely introduced PDMS and mPEG chains, using P(EO/PO/AGE) with higher AGE content as starting materials.

## Figures and Tables

**Table 1:** PDMS content, *T*_c_, and *T*_m_ of PDMS & mPEG-g-P(EO/PO/AGE)s.

Polymer	PDMS Content (wt%)	*T*_c_ (°C)	*T*_m_ (°C)
25%PDMS & 75%mPEG1K-g-P(EO/PO/AGE)	9	33	50
25%PDMS & 75%mPEG2K-g-P(EO/PO/AGE)	7	34	59
25%PDMS & 75%mPEG4K-g-P(EO/PO/AGE)	5	40	59
50%PDMS & 50%mPEG1K-g-P(EO/PO/AGE)	18	32	51
50%PDMS & 50%mPEG2K-g-P(EO/PO/AGE)	15	33	53
50%PDMS & 50%mPEG4K-g-P(EO/PO/AGE)	11	36	58
75%PDMS & 25%mPEG1K-g-P(EO/PO/AGE)	27	31	49
75%PDMS & 25%mPEG2K-g-P(EO/PO/AGE)	25	32	51
75%PDMS & 25%mPEG4K-g-P(EO/PO/AGE)	21	34	53

**Table 2:** Preparation of PDMS-*g*-P(EO/PO/AGE)s.

Polymer	P(EO/PO/AGE) (g)	PDMS-SiH (g)	Yield (%)
25%PDMS-g-P(EO/PO/AGE)	6.0	0.7	93
50%PDMS-g-P(EO/PO/AGE)	6.0	1.4	95
75%PDMS-g-P(EO/PO/AGE)	6.0	2.1	96

**Table 3:** Preparation of PDMS & mPEG-g-P(EO/PO/AGE)s.

Polymer	PDMS-*g*-P(EO/PO/AGE) (g)	mPEG-N_3_ (g)	Yield (%)
25% PDMS & 75% mPEG1K-g-P(EO/PO/AGE)	25% PDMS-*g*-P(EO/PO/AGE) (1.0)	mPEG1K-N3 (0.40)	85
25% PDMS & 75% mPEG2K-g-P(EO/PO/AGE)		mPEG2K-N3 (0.80)	83
25% PDMS & 75% mPEG4K-g-P(EO/PO/AGE)		mPEG4K-N3 (1.6)	82
50% PDMS & 50% mPEG1K-g-P(EO/PO/AGE)	50% PDMS-*g*-P(EO/PO/AGE) (1.0)	mPEG1K-N3 (0.24)	51
50% PDMS & 50% mPEG2K-g-P(EO/PO/AGE)		mPEG2K-N3 (0.48)	53
50% PDMS & 50% mPEG4K-g-P(EO/PO/AGE)		mPEG4K-N3 (0.96)	58
75% PDMS & 25% mPEG1K-g-P(EO/PO/AGE)	75% PDMS-*g*-P(EO/PO/AGE) (1.0)	mPEG1K-N3 (0.11)	80
75% PDMS & 25% mPEG2K-g-P(EO/PO/AGE)		mPEG2K-N3 (0.22)	82
75% PDMS & 25% mPEG4K-g-P(EO/PO/AGE)		mPEG4K-N3 (0.44)	94

## Data Availability

The original contributions presented in this study are included in the article. Further inquiries can be directed to the corresponding author.

## Abbreviations

The following abbreviations are used in this manuscript:

PEO	Polyethylene oxide
SPE	Solid polymer electrolyte
EO	Ethylene oxide
PO	Propylene oxide
AGE	Allyl glycidyl ether
PDMS	Polydimethylsiloxane
mPEG	Polyethylene glycol monomethyl ether
LiTFSI	Lithium bis(trifluoromethanesulfonyl)imide
